# KLF7 Promotes Hepatocellular Carcinoma Progression Through Regulating SLC1A5‐Mediated Tryptophan Metabolism

**DOI:** 10.1111/jcmm.70245

**Published:** 2024-12-08

**Authors:** Bao Chai, Anhong Zhang, Yang Liu, Xi Zhang, Pengzhou Kong, Zhuowei Zhang, Yarong Guo

**Affiliations:** ^1^ Department of Gastroenterology, Shanxi Bethune Hospital, Shanxi Academy of Medical sciences, TongilShanxi Hospital Third Hospital of Shanxi Medical University Taiyuan Shanxi China; ^2^ Department of Surgery The First Affiliated Hospital of Shanxi Medical University Taiyuan Shanxi China; ^3^ Shanxi Medical University Taiyuan Shanxi China; ^4^ Translational Medicine Research Center, Key Laboratory of Cellular Physiology (Shanxi Medical University), Ministry of Education, Department of Pathology Shanxi Medical University Taiyuan Shanxi China; ^5^ College of Medical Imaging Shanxi Medical University Taiyuan Shanxi China; ^6^ Department of Digestive System Oncology, Shanxi Bethune Hospital, Shanxi Academy of Medical sciences, TongilShanxi Hospital Third Hospital of Shanxi Medical University Taiyuan Shanxi China; ^7^ Department of Oncology The First Affiliated Hospital of Shanxi Medical University Taiyuan Shanxi China

**Keywords:** HCC, KLF7, SLC1A5, tryptophan metabolism

## Abstract

Krüppel‐like factor 7 is a transcriptional activator and acts as an oncogene in human cancers, including hepatocellular carcinoma (HCC). Tryptophan metabolism is important for HCC cell proliferation, metastasis, and invasion. It is unclear whether KLF7 could regulate Trp metabolism in HCC. In this study, we found that Trp metabolism was suppressed in HCC cells with KLF7 knockdown. The mRNA and protein levels of SLC1A5, SLC7A5, and TPH1, as well as the content of Trp and serotonin, were reduced after KLF7 knockdown, and were potentiated following KLF7 overexpression. Increasing the content of serotonin could restore the malignancy of tumour cells in vitro and tumour growth in vivo. Conversely, decreasing the content of serotonin suppressed HCC cell proliferation. The binding activity of KLF7 was on the promoter of SLC1A5, and KLF7 positively regulated the expression of SLC1A5. KLF7 contributed to the proliferation and migration of HCC cells by up‐regulation of SLC1A5. Collectively, KLF7 promotes the progression of HCC through regulating Trp metabolism. The newly identified axis of KLF7/ SLC1A5 in HCC could represent a potential target for HCC therapy.

**Abbreviations**
DEG: differentially expressed genes
DT: double tryptophan
HCC: hepatocellular carcinoma
KLF7: Krüppel‐like factor 7
RNA‐seq: RNA sequencing
SLC1A5: solute carrier family 1 member 5
SLC7A5: solute carrier family 7 member 5
TDO2: Trp 2,3‐dioxygenase
TPH1: Trp hydroxylase 1
Trp: Tryptophan
5‐HT: 5‐hydroxytryptamine
5‐HTP: L‐5‐hydroxytryptophan

## 1 Introduction

Hepatocellular carcinoma (HCC) is the most common primary malignancy of the liver and is often associated with chronic liver disease and cirrhosis [1, 2]. Survival in patients with HCC is determined by the anatomic phase, biologic grade, and severity of the cirrhosis [2]. Although medical, locoregional, and surgical therapies have advanced rapidly, HCC remains one of the most common causes of cancer‐associated deaths worldwide [3]. There will be estimated one million annual deaths from HCC by 2030 [4]. Patients with advanced HCC have few treatment options and a poor prognosis [5]. The relative 5‐year survival rate is approximately 18% [4]. With regard to its clinical and molecular characteristics, HCC is a very heterogeneous cancer, which explains why predictive biomarkers of response to HCC therapy have not been identified to date [6]. Identifying credible predictive biomarkers could benefit the diagnosis and treatment of the patients.

Krüppel‐like factors (KLFs), a class of highly conserved zinc fingers that include transcription elements, have important physiological and pathological functions [7]. KLFs exhibit multiple roles in the pathogenesis of cancers and could be considered valuable prognostic factors and hidden biological targets for the diagnosis and treatment of cancer patients [8]. Krüppel‐like factor 7 (KLF7) is classified as a transcriptional activator [9], and acts as an oncogene in lung adenocarcinoma [10], gastric cancer [11], head and neck squamous cell carcinoma [12], pancreatic ductal adenocarcinoma [13], glioma [14], ovarian cancer [15], and osteosarcoma [16].

Tryptophan (Trp) is an essential amino acid which is acquired by food intake. The majority of trp is metabolised through kynurenine (Kyn) pathway, while the remaining can be the substrate for 5‐Hydroxytryptophan and indole pathways [17]. Trp and its metabolites are important for the physiologic activities, such as proliferation, survival and cell death in normal cells. Previous studies showed that trp metabolism was involved in the progression of various malignancies. For example, elevated expression of SLC1A5 and SLC7A5 which regulate the transport of trp was obviously associated with the poor prognosis of several cancers, including lung, kidney, skin and breast cancers [18]. In addition, up‐regulation of SLC1A5 and SLC7A5 potentiated the uptake of try and intracellular metabolism of kynurenine pathway to promote the progression of Myc‐induced colon cancer [19]. These studies suggest that SLC1A5 and SLC7A5‐mediated trp intake supports the malignant growth of tumour cells. However, the significance of KLF7 in trp metabolism is poorly understood.

In HCC, our previous research showed the overexpression of KLF7 in HCC, and KLF7 drove tumour development and cell invasion of HCC by binding to the promoter of Ccdc85c [20]. KLF7 could be a target gene for miR‐370‐5P and miR‐186‐5p in the tumorigenesis of HCC, and down‐regulation of KLF7 suppressed the malignant progression of HCC. These reports confirmed the proposed oncogenic role of KLF7 in HCC [21, 22]. In this study, we intended to explore the molecular events during KLF7‐promoted HCC development. We identify a new molecular axis and a suitable metabolic characteristic in HCC triggered by KLF7.

## 2 Materials And Methods

### 2.1 Human HCC Specimens and IHC Staining

Human HCC samples were collected from the patients who underwent surgery at Shanxi Bethune Hospital between 2020 and 2024. A total of 30 patients were included in this study. This study was approved by The First Affiliated Hospital of Shanxi Medical University (2022‐K‐K0047). An informed consent was signed by each patient. The samples were fixed in 4% paraformaldehyde and subjected to IHC staining of KLF7 and SLC1A5, according to the protocols as described previously [23, 24]. KLF7 primary antibody was purchased from abcam (1:100, ab197690). SLC1A5 primary antibody was obtained from Proteintech (Cat no. 20350‐1‐AP).

### 2.2 Cell Cultures and Regents

SKHEP1 and Huh7 cells were obtained from Procell (Wuhan, China) and cultured in Dulbecco's modified Eagle medium (Thermo Fisher Scientific) supplemented with 10% fetal bovine serum (Gibco), 100 mU/mL penicillin and 100 μg/mL streptomycin (Sigma). Cells were maintained in 37°C incubator with a 5% CO_2_ atmosphere. Sertraline was provided by MedChemExpress (USA). Serotonin was provided by Sigma (USA). DMEM/F‐12 medium lacking tryptophan (Trp) was purchased from Biologicals (USA).

### 2.3 Cell Transfection and Infection

RIBOBIO (China) designed and synthesised short interfering RNAs targeting KLF7. The sequences of the siRNAs used in this study were: siKLF7‐1: 5′‐GGGUUCUUCCAAAGAACAUdTdT‐3′, siKLF7‐2: 5′‐GGGCGUACCATGGGAUUAUAUdTdT‐3′, and siCtrl: 5′‐CAGUACUUUUGUGUAGUACAAAdTdT‐3′. The short hairpin RNA for KLF7 was subcloned into the pRNA‐H1.1 plasmid. The sequences of shRNAs were: shCtrl: 5′‐UUCTCCGAACGUGUCACGU‐3′, shKLF7‐1: 5′‐GCUUCUCAGUGCAACUGAUA‐3′, and shKLF7‐2: 5′‐GGGCGUACCATGGGAUUAUAU‐3′. Lentivirus was packaged in 293FT cells. shCtrl and shKLF7 stable cell lines were constructed by infecting the cells with lentivirus and selected by neomycin. For KLF7 and SLC1A5 overexpression, the coding sequence of KLF7 and SLC1A5 was inserted into pcDNA3.1 expression vectors (Clontech, USA), respectively. The promoter (−2000 bp) of the SLC1A5 gene was synthesised and subcloned into the pGL3. Basic vector for the luciferase assay. Transfection was conducted using Lipofectamine 3000.

### 2.4 RNA Sequencing

Total RNA from Huh7 cells that were transfected with siCtrl or siKLF7 for 24 h was extracted using TRIzol reagent. Randomly cleaved into short template fragments of 200–500 bp from each RNA sample were reversed‐transcribed to produce a cDNA library. A 2 × 150 paired‐end sequencing protocol was adopted to sequence all cDNA libraries on the Illumina HiSeq 2000 high‐throughput system, with the original sequencing information in FASTQ format. Reads of each sample with the human reference genome were aligned with TopHat v1.4.1. The “DESeq2” package of the R software was used to detect differentially expressed genes (DEG) in cells, with a |log 2‐fold change| (|log2FC|) ≥ 0.6 and modified *p* < 0.05 as the threshold values. The enrichment pathways associated with DEGs were explored and the Kyoto Encyclopedia of Genes and Genomes was used for the analysis.

### 2.5 Metabonomics Analysis

Huh7 cells transfected with siCtrl or siKLF7 for 24 h were collected and maintained at—80°C until analysis. Samples were sent to Metware Biotechnology CO. Ltd. (Wuhan, Chian) for metabonomics analysis.

### 2.6 Real‐Time PCR

Total RNA from transfected HCC cells was extracted with TRIzol, and was subjected to reversed transcription with Superscript II. Real‐time PCR assay was performed using SYBR Premix Ex Taq on a 7500 real‐time PCR system. The 2(−ΔΔCT) approach was adopted to calculate relative expression of mRNA, normalising to β‐actin mRNA levels. The primers were synthesised by HippoBio (Huzhou, China), and the sequences were as follows: SLC1A5, forward, 5′‐TCATGTGGTACGCCCCTGT‐3′, reverse, 5′‐GCGGGCAAAGAGTAAACCCA‐3′; SLC7A5, forward, 5′‐CCGTGAACTGCTACAGCGT‐3′, reverse, 5′‐CTTCCCGATCTGGACGAAGC‐3′; TPH1, forward, 5′‐ACGTCGAAAGTATTTTGCGGA‐3′, reverse, 5′‐ACGGTTCCCCAGGTCTTAATC‐3′; and β‐actin, forward, 5′‐CATGTACGTTGCTATCCAGGC‐3′, reverse, 5′‐CTCCTTAATGTCACGCACGAT‐3′.

### 2.7 Western Blotting

The proteins were extracted from transfected HCC cells with RIPA lysis buffer, supplemented with a protease and phosphatase inhibitor cocktail (P1046, Beyotime). TaKaRa BCA protein assay kit was used to assess protein concentration. Next, 10% SDS‐PAGE was used to separate the total protein. After transferring onto PVDF membranes (MBS2557001, MyBiosource, San Diego, United States), the membranes were blocked in TBS buffer containing 5% BSA. The following primary antibodies were used: anti‐KLF7 (1:1000, ab197690, abcam), anti‐SLC1A5/ASCT2 (1:1000, ab187692, abcam), anti‐SLC7A5/LAT1 (1:1000, ab99419, abcam), anti‐TPH1 (1:1000, MBS8504031, MyBioSource), and anti‐GAPDH (1:1000, ab263962, abcam). Lastly, the membranes were incubated with the goat anti‐rabbit IR680 secondary antibody, followed by imaging with a LI‐COR OdysseySA imaging system.

### 2.8 Enzyme‐Linked Immunosorbent Assay

SKHEP1 and Huh7 cells were seeded in 24‐well plates at 1 × 10^5^ cells/cm^2^ for 24 h and the cells were collected and saved at −80°C. The concentrations of Trp and 5‐hydroxytryptamine (serotonin) in transfected HCC cells were measured using the Serotonin enzyme‐linked immunosorbent assay (ELISA) kit (ELK7830, ELK Biotechnology) and the Trp Assay Kit (ELK8262), according to the manufacturers' instructions.

### 2.9 Cell Proliferation and Migration Assays

To determine cell viability, cell count kit‐8 (CCK8) was used. Transfected cells treated with drugs were cultured for 24, 48, 72, and 96 h. The CCK8 reagent was then added into each well and incubated for 4 h at 37°C. The absorbance measurement was performed at 450 nm.

For the cell colony formation assay, transfected cells were seeded in six‐well plates, culturing at 37°C for 7–14 days. The medium was changed every 3 days. The cells were then fixed in paraformaldehyde (4%), dyed with 0.5% crystal violet, and imaged with a digital camera.

For migration assay, 6 × 10^5^ SKHEP1 and Huh7 cells treated as described above were seeded in the upper chamber of the Transwell chamber in serum‐free DMEM medium and the complete DMEM culture medium was placed in the lower compartment. The cell culture was incubated for 24 h at 37°C, and then, cotton swabs were used to gently scrape off the cells on the upper side of the Transwell membrane. Fixed with 4% paraformaldehyde, cell inserts were stained with 0.1% crystal violet for 15 min at room temperature, washed three times with PBS, and then photographed.

### 2.10 Animal Models

Breeding and housing of the animals were performed in the absence of pathogen. All animal tests were carried out according to the guidelines of the Institutional Animal Care and Use Committee of The First Affiliated Hospital of Shanxi Medical University (SYXK2019‐0007). Twenty‐five female BALB/c nude mice (4–5 weeks‐old) were provided by Beijing Charles River. A total of 1 × 10^6^ SKHEP1 cells was subcutaneously injection into the right flank of mice. Once palpable tumours were formed, mice bearing shCtrl and shKLF7 tumours were randomly distributed into four groups (*n* = 5), including the control plus normal trp group (shCtrl + Vehicle), shCtrl plus double trp group (shCtrl + DT), shKLF7 plus normal trp group (shKLF7 + Vehicle), and the shKLF7 plus double trp (shKLF7 + DT) group. The non‐transfected cell implantation control group was also included. All of the mice were fed with Trp‐free diet and with Vehicle (2 mg/kg Trp) or DT (4 mg/kg Trp) water. Serial measurements of tumour volumes were performed every 3 days. Nineteen days later, the mice were sacrificed and the tumours were collected for further tests.

### 2.11 Chromatin Immunoprecipitation‐qPCR Assay

To examine whether KLF7 interacted with the promoter of SLC1A5, we amplified the coding sequence of KLF7 into pCDNA3.1 vectors, containing the Flag. Then SKHEP1 and Huh7 cells were transfected with pCDNA3.1/KLF7‐Flag vectors for 48 h. The cells were subjected to chromatin immunoprecipitation assay with IgG and Flag antibody using SimpleChIP enzymatic chromatin IP kit (Cell Signalling), following the manufacturer's protocols. The relative enrichment of SLC1A5 promoter sequence was detected by qPCR assay in both groups.

### 2.12 Luciferase Reporter Assay

After transfecting SKHEP1 and Huh7 cells with siRNAs, overexpressing vectors of KLF7, as well as the pGL3. Basic and TK vectors, cells were cultured for 24 h and lysed. Dual luciferase activity was assessed with the kit from Promega on the POLARStar Omega plate reader.

### 2.13 Statistical Analysis

Data are shown as mean ± standard deviation. Statistical analyses were performed with GraphPad Prism 7 (GraphPad Software). The comparison between two groups was made using the students t‐test. ANOVA analysis of variance was adopted to measure the differences among more than two groups. *p* ≤ 0.05 considered statistically significant.

## 3 Results

### 3.1 Tryptophan Metabolism Is Regulated by KLF7 in HCC

**FIGURE 1:**
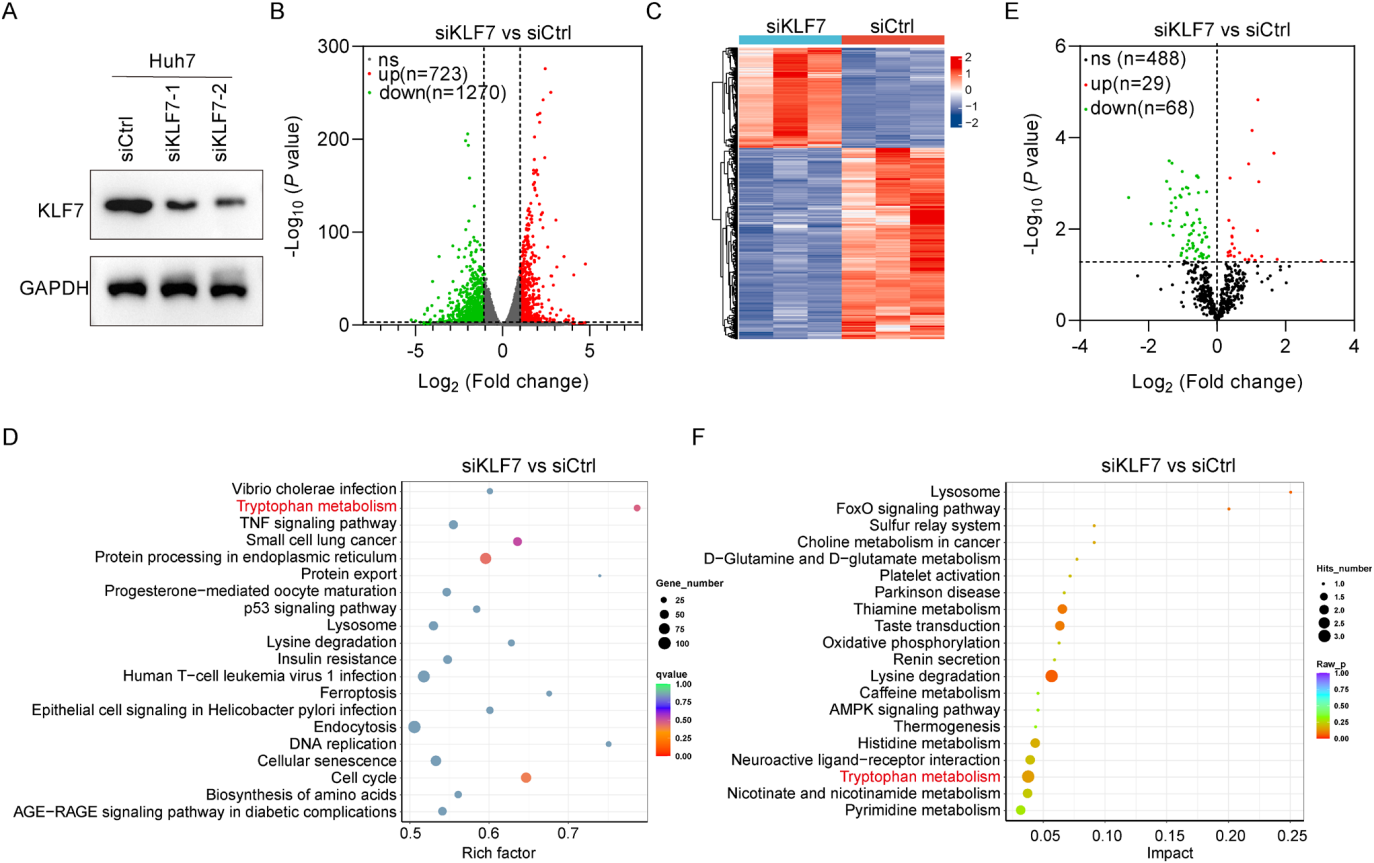
Tryptophan metabolism enriches in KLF7‐regulated HCC progression. (A) Immunoblotting analysis of KLF7 and GAPDH in siCtrl, siKLF7‐1 and siKLF7‐2 Huh7 cells. (B and C) The heatmap results and volcano map showing differentially expressed genes (DEG)s in Huh7 cells transfected with siRNAs against KLF7 (siKLF7) using RNA‐seq. (D) KEGG enrichment analyses of genes differentially expressed after KLF7 knockdown. (E) The volcano map of differentially expressed metabolites in siKLF7 transfected Huh7 cells based on metabonomic analysis. (F) KEGG enrichment analyses of metabonomic signalling after KLF7 downregulation.

To identify the target genes or pathways involved in KLF7‐triggered HCC development, we performed RNA sequencing (RNA‐seq) in Huh7 cells transfected with siRNAs against negative control (siCtrl) and KLF7 (siKLF7). Firstly, the knockdown efficacy of KLF7 was validated by immunoblotting assay (Figure 1A). We subjected siCtrl and siKLF7 cells to RNA‐seq analysis. The heatmap and volcano results showed that 723 genes were significantly up‐regulated, while 1270 genes were markedly decreased in KLF7 knockdown Huh7 cells (Figure 1B,C). DEGs were subjected to KEGG enrichment analyses, revealing that Trp metabolism signalling was significantly dysregulated following KLF7 downregulation (Figure 1D). Therefore, we also performed a metabonomic analysis in Huh7 cells with knockdown of KLF7. The results identified 97 differentially expressed metabolites, including 29 upregulated metabolites and 68 down‐regulated metabolites (Figure 1E). KEGG analyses also identified enriched Trp metabolism pathways (Figure 1F). We hypothesized that Trp metabolism may be involved in KLF7‐regulated HCC progression.

### 3.2 KLF7 Influences the Expression of Genes Involved in Tryptophan Metabolism

**FIGURE 2:**
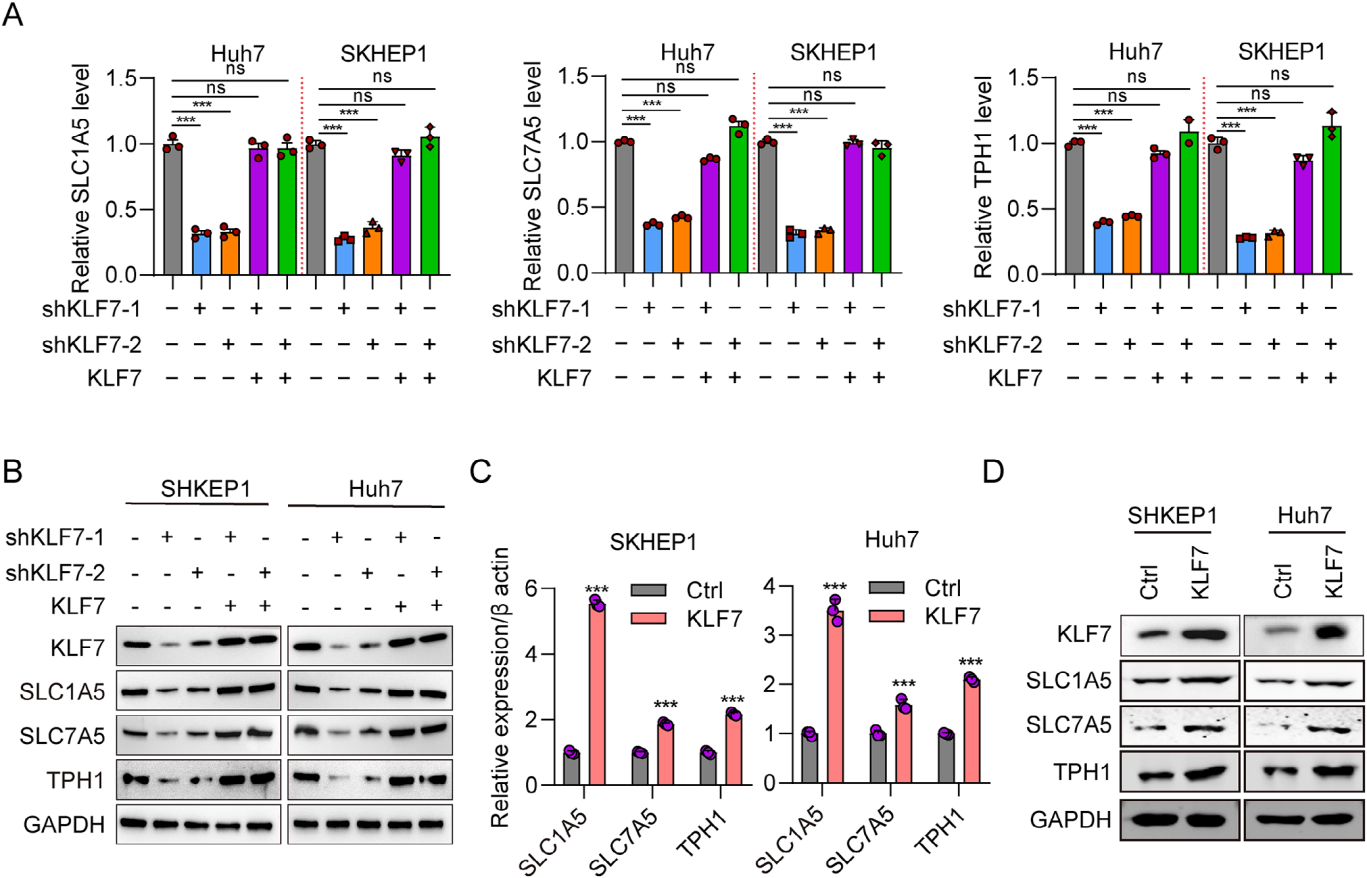
KLF7 Potentiates the expression of tryptophan metabolism markers. (A) Real‐time PCR was performed in two HCC cell lines (SKHEP1 and Huh7) transfected with shCtrl, shKLF7‐1, shKLF7‐2, shKLF7‐1 + KLF7 and shKLF7‐2 + KLF7 to quantify of the transcription levels of SLC1A5, SLC7A5, and TPH1. β‐Actin was used as an internal reference gene. ***p < 0.001 and ns (not significant) versus shCtrl. (B) Western blotting was performed in two HCC cell lines to quantify the expression of the SLC1A5, SLC7A5, and TPH1 proteins. The intensity of the protein bands was normalised to GAPDH. (C) Real‐time PCR analysis of SLC1A5, SLC7A5, and TPH1 mRNA levels in HCC cell lines transfected with Ctrl and KLF7. ***p < 0.001 versus Ctrl. (D) Protein levels of SLC1A5, SLC7A5, and TPH1 in HCC cell lines transfected with Ctrl and KLF7. GAPDH was used to normalised protein levels. All experiments were performed at least three times.

Trp, an essential amino acid, is transported to cells by the membrane amino acid transports solute carrier family 1 member 5 (SLC1A5) and solute carrier family 7 member 5 (SLC7A5) [25, 26]. Trp hydroxylase 1 (TPH1), one isoform of TPHs which transform Trp to serotonin, plays a pivotal role in Trp metabolism [27]. To explore Trp associated genes regulated by KLF7, the mRNA and protein levels of SLC1A5, SLC7A5, and TPH1 were evaluated in HCC cells transfected with shKLF7 and shCtrl. The results of RT‐qPCR showed that SLC1A5, SLC7A5, and TPH1 transcripts in SKHEP1 and Huh7 cells were significantly downregulated after KLF7 knockdown (shKLF7‐1 and shKLF7‐2) compared to controls (Figure 2A). Western blotting demonstrated the knockdown efficiency of KLF7 in shKLF7‐1 and shKLF7‐2 SKHEP1 and Huh7 cells (Figure 2B). The results also showed strong signals of SLC1A5, SLC7A5, and TPH1 in SKHEP1 and Huh7 cells transfected with shCtrl and a reduced signal in cells transfected with shKLF7‐1 and shKLF7‐2 (Figure 2B). Rescue experiments indicated that restoring the expression of KLF7 up‐regulated the mRNA level of SLC1A5, SLC7A5, and TPH1 in shKLF7‐1 and shKLF7‐2 cells to that in shCtrl cells (Figure 2A,B). We also constructed an overexpression plasmid of KLF7 to increase the ectopic expression of KLF7. RT‐PCR and western blotting showed that both mRNA and protein levels of SLC1A5, SLC7A5, and TPH1 were significantly induced after KLF7 overexpression (Figure 2C,D). These results showed that KLF7 could affect the expression of the crucial genes involved in Trp metabolism.

### 3.3 KLF7 Influences the Levels of Tryptophan and 5‐HT in Hepatoma Cells

**FIGURE 3:**
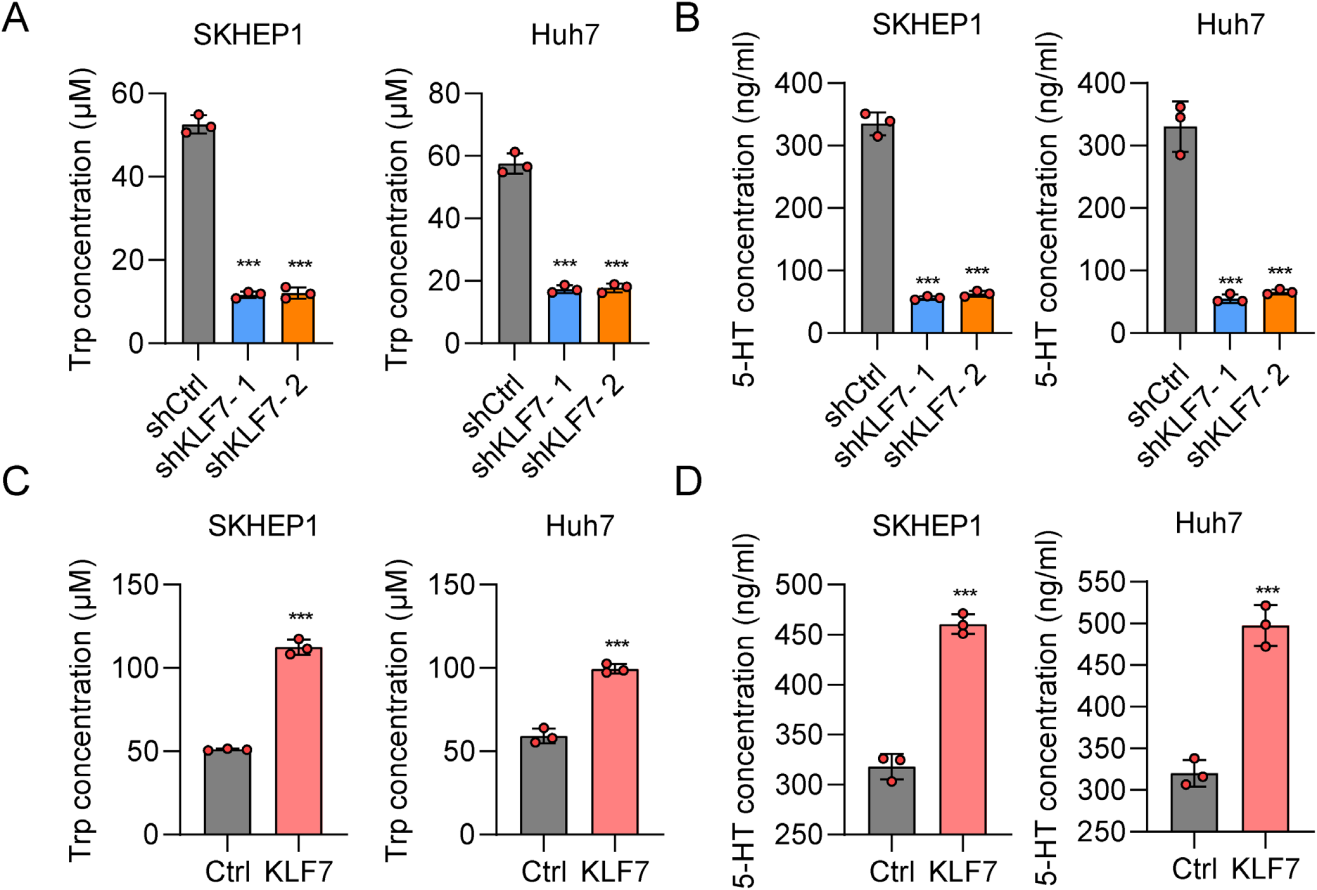
KLF7 affects the content of tryptophan and 5‐HT in HCC cells. (A, B) ELISA assay was performed to determine the levels of Trp (A) and 5‐HT (B) in SKHEP1 and Huh7 cells that were transfected with shCtrl, shKLF7‐1 and shKLF7‐2. ***p < 0.001 versus shCtrl. (C, D) ELISA assay was performed to determine the concentration of Trp (C) and 5‐HT (D) in SKHEP1 and Huh7 cells that were transfected with Ctrl and KLF7. ***p < 0.001 versus Ctrl. All experiments were performed at least three times.

Our findings showed that KLF7 regulates the expression of Trp metabolism markers, whereas it is not clear whether Trp levels are also influenced. Trp is converted to L‐5‐hydroxytryptophan by tryptophan hydroxylase, and 5‐HTP decarboxylation produces serotonin [27]. Therefore, we measured the concentration of Trp and 5‐HT in HCC cells after KLF7 knockdown or overexpression using a commercial ELISA kit. The results showed that Trp and 5‐HT content decreased significantly in SKHEP1 and Huh7 cells after KLF7 knockdown (Figure 3A,B), while they increased significantly after KLF7 overexpression (Figure 3C,D). These results showed that KLF7 regulated Trp and 5‐HT levels in HCC cells.

### 3.4 KLF7 Regulates the Malignant Phenotype of Hepatocellular Carcinoma Through Potentiating the Metabolism of Tryptophan

**FIGURE 4:**
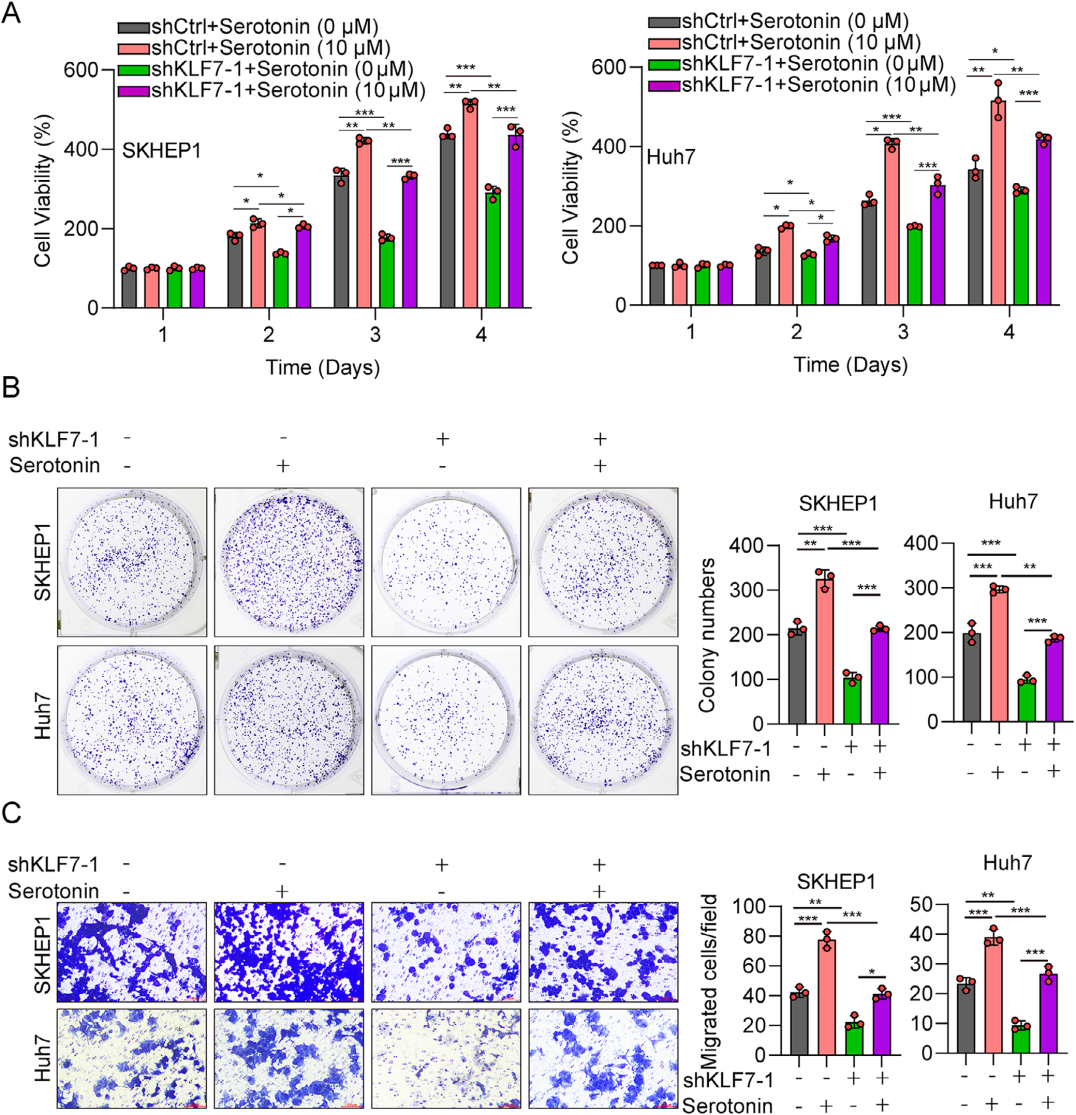
KLF7 knockdown inhibits the malignant phenotype of hepatocellular carcinoma through tryptophan metabolism. (A) The proliferation of SKHEP1 and Huh7 cells transfected with shCtrl or shKLF7 and treated with serotonin (10 μM) was evaluated by the CCK‐8 assay. (B) The colony generation capacity of SKHEP1 and Huh7 cells as described in A. (C) The migration ability of SKHEP1 and Huh7 cells as described in A was detected by Transwell assay. *p < 0.05, **p < 0.01, ***p < 0.001. All experiments were performed at least three times.

**FIGURE 5:**
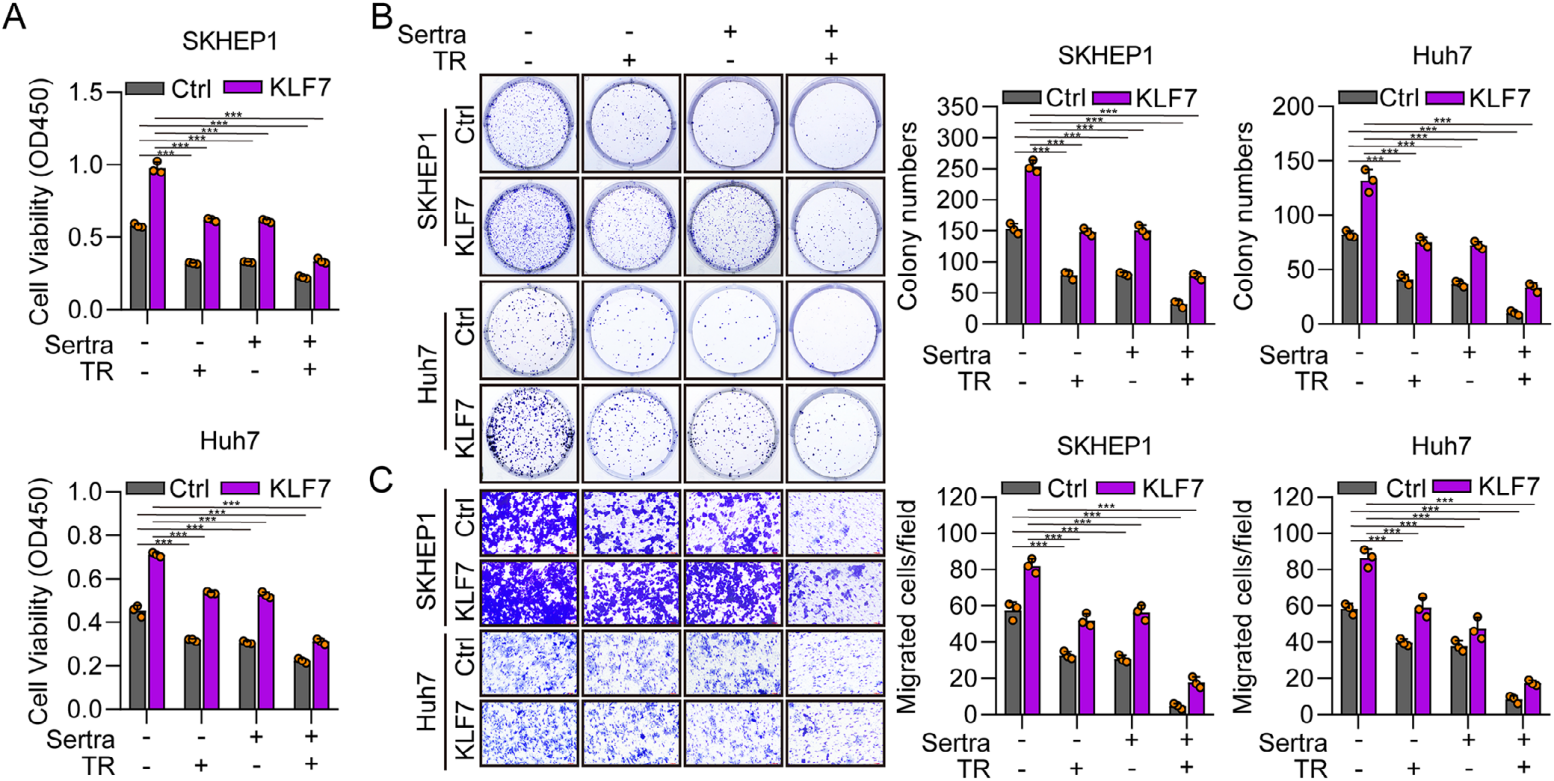
KLF7 overexpression promotes the malignant phenotype of hepatocellular carcinoma through the metabolism of tryptophan. (A) The proliferation of SKHEP1 and Huh7 cells transfected with Ctrl or KLF7 expression plasmids and treated with sertraline (10 μM) or cultured in DMEM/F‐12 without Trp was determined by the CCK‐8 assay. (B, C) The colony formation (B) and migration (C) capacity of SKHEP1 and Huh7 cells as described in A. ***p < 0.001. Sertra, sertraline; TR, DMEM/F‐12 without Trp. All experiments were performed at least three times.

To explore whether KLF7 promotes HCC proliferation and migration through Trp metabolism, we applied serotonin and a selective 5‐HT uptake inhibitor, sertraline [28] to treat two HCC cell lines. The CCK‐8 results showed that shKLF7 alone significantly decreased the viability of SKHEP1 and Huh7 cells, while serotonin treatment markedly increased the propagation of HCC cells (Figure 4A). The cell viability of cells exposed to shKLF7 plus serotonin was higher than that of the shKLF7 group and was lower than that of the serotonin group (Figure 4A), indicating that serotonin could overcome the suppressed function of KLF7 knockdown in cell proliferation. Cell colony formation assay and the Transwell assay obtained similar results (Figure 4B,C). Conversely, we also found that KLF7 overexpression could greatly increase the proliferation of HCC cells, while sertraline‐treated cells obviously decreased cell viability and cell colony formation (Figure 5A,B). Furthermore, transfected cells cultured in DMEM F‐12 lacking Trp slowed the cell proliferation capacity (Figure 5A,B). Consistent results were observed in Transwell assay (Figure 5C). These results suggested that KLF7 regulated the progression of HCC through Trp metabolism.

### 3.5 KLF7 Regulates Hepatocellular Carcinoma Tumour Growth Through Tryptophan Metabolism

**FIGURE 6:**
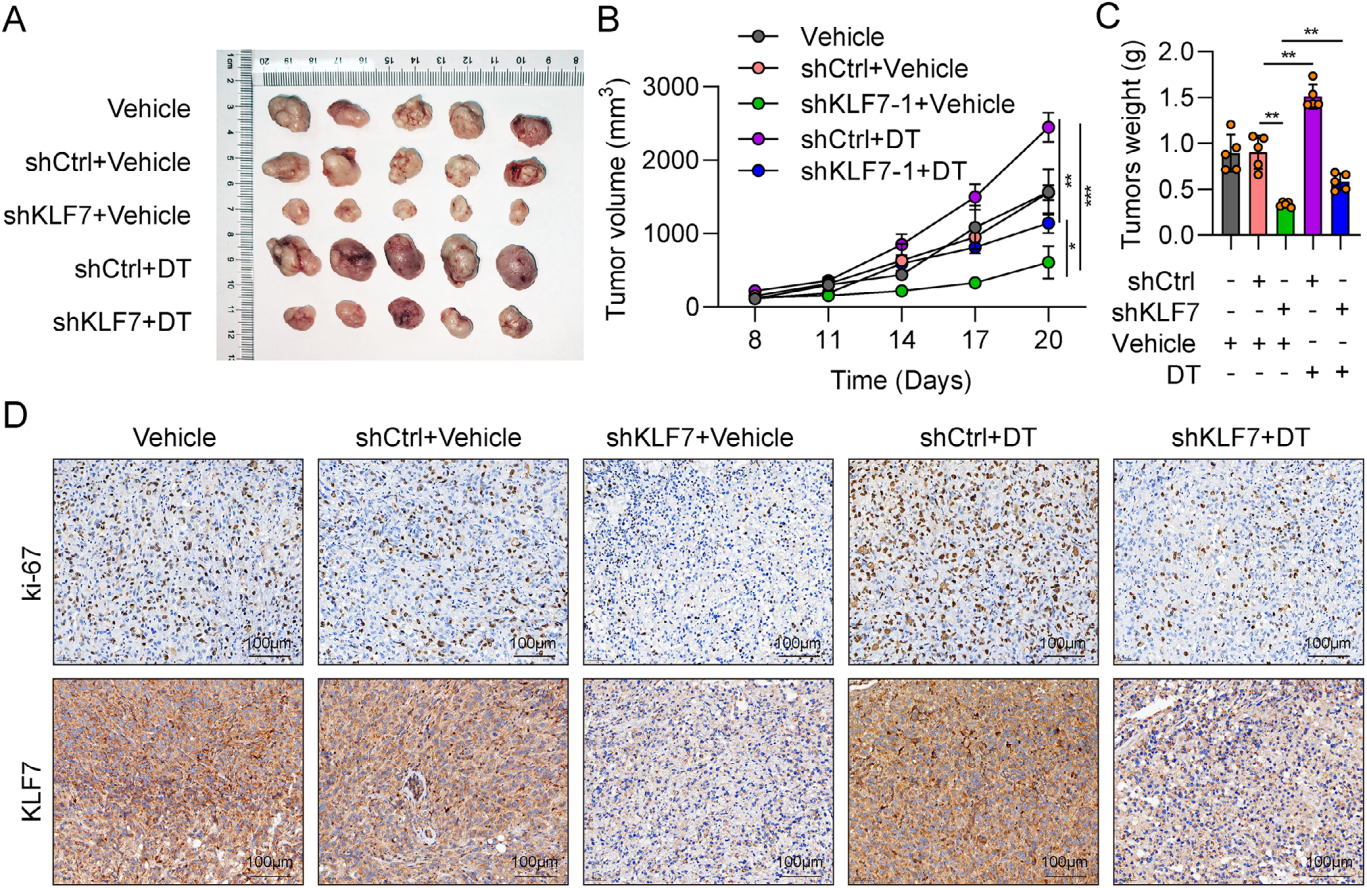
KLF7 contributes to tumour growth of hepatocellular carcinoma through tryptophan metabolism. (A) The macroscopical images of the tumours, *n* = 5. (B) Tumour volumes monitored over time in the non‐transfected group and the four treatment groups of BALB/c mice. (C) Tumour weight of BALB/c mice in the non‐transfected group and the four treatment groups. (D) IHC staining of Ki‐67 and KLF7 in the tumour tissues as shown in A. Scale bar, 100 μm. All of the mice were fed with Trp‐free diet and with Vehicle (2 mg/kg Trp) or DT (4 mg/kg Trp) water. *p < 0.05, **p < 0.01, ***p < 0.001.

To determine whether KLF7 promotes HCC tumorigenesis through Trp metabolism, we established a tumour xenograft model. Mice were injected with HCC cells transfected with shCtrl or shKLF7, combined with diets that included double tryptophan (DT). In addition, non‐transfected cells were also implanted into the mice to exclude the effect of lentivirus on the xenografted tumour development of SKHEP1 cells. As shown in Figure 6, the impact of lentivirus on the tumorigenesis of SKHEP1 cells in vivo was not observed (Vehicle vs. shCtrl + Vehicle). Compared with the control group (shCtrl + Vehicle), mice in the DT group (shCtrl + DT) exhibited significantly increased average tumour size, volume, and weight (Figure 6A–C), indicating that DT promoted HCC tumour growth. Compared to the control group, the KLF7 downregulation (shKLF7 + Vehicle) greatly decreased tumour growth, suggesting that silencing of KLF7 suppressed HCC tumorigenesis (Figure 6A–C). Furthermore, we found that in the shKLF7 plus DT group (shKLF7 + DT) showed a significantly increased tumour size, volume, and weight compared to the shKLF7 group (shKLF7 + Vehicle), revealing that DT could restore the tumour suppressive effect of KLF7 knockdown on the progression of HCC (Figure 6A–C). In addition, we subjected the tumour tissues to IHC staining of KLF7 and Ki‐67. KLF7 was significantly reduced in the tumours of shKLF7‐1 + Vehicle and shKLF7‐1 + DT as compared with other tumours (Figure 6D). Ki‐67 results suggested that the numbers of proliferative cells were highest in shCtrl+DT tumours and lowest in shKL7 + Vehicle tumours, while were moderate in other tumours (Figure 6D). The results of Ki‐67 expression were consistent with those of the tumour growth curves and tumour weights. In conclusion, we suggest that KLF7 regulates the growth of HCC tumours through Trp metabolism.

### 3.6 KLF7 Regulates Tryptophan Metabolism by Targeting SLC1A5

**FIGURE 7:**
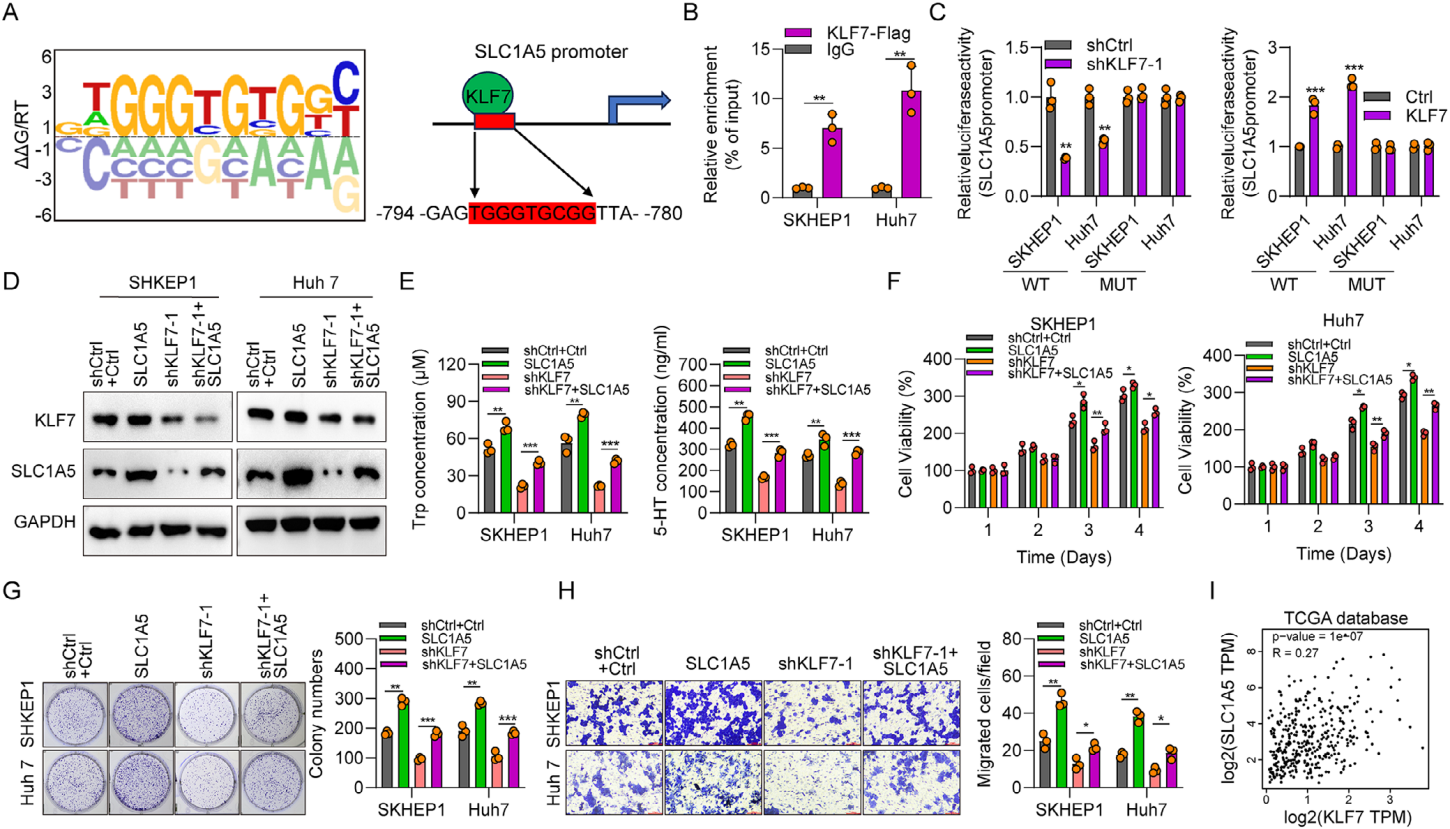
KLF7 regulates tryptophan metabolism by targeting SLC1A5. (A) The predictive binding sequence of SLC1A5 promoter for KLF7. (B) ChIP‐qPCR assay was performed to verify the interaction between KLF7 and the promoter of SLC1A5. (C) Luciferase reporter assays were performed in SKHEP1 and Huh7 cells carrying the vectors containing widetype (WT) or mutated (MUT) SLC1A5 promoter region and KLF7 expression plasmids or shKLF7‐1. After 24 h of transfection. The relative activity of luciferase was determined. (D) Western blotting showing KLF7 and SLC1A5 expression in SKHEP1 and Huh7 cells transfected with shCtrl, shCtrl+SLC1A5, shKLF7, and shKLF7 + SLC1A5. (E) ELISA was conducted to measure Trp and 5‐HT content in SKHEP1 and Huh7 cells transfected with shCtrl, shCtrl+SLC1A5, shKLF7, and shKLF7 + SLC1A5. (F) The proliferation of SKHEP1 and Huh7 cells transfected with shCtrl, shCtrl+SLC1A5, shKLF7, and shKLF7 + SLC1A5 was evaluated by CCK‐8 assay. (G) The colony generation capacity of SKHEP1 and Huh7 cells transfected with shCtrl, shCtrl + SLC1A5, shKLF7, and shKLF7 + SLC1A5. (H) The Transwell assay was performed to determine cell migration of SKHEP1 and Huh7 cells transfected with shCtrl, shCtrl + SLC1A5, shKLF7, and shKLF7 + SLC1A5. (I) The association between KLF7 and SLC1A5 expression in HCC samples derived from TCGA database. *p < 0.05, **p < 0.01, ***p < 0.001.

Lastly, we investigated the mechanisms underlying the role of KLF7 in Trp metabolism. The predictive binding site of KLF7 on the promoter of SLC1A5, SLC7A5 and TPH1 genes was shown in Figure 7A and Figure S1. We predict that KLF7 may increase the transcription activity of these three genes via direct interaction. To validate whether KLF7 transcriptionally activates SLC1A5, we performed ChIP‐qPCR and luciferase assays. The ChIP‐qPCR assay confirmed the interaction between KLF7 and SLC1A5's promoter (Figure 7B). Luciferase reporter results demonstrated that KLF7 enhanced the transcription activity of SLC1A5 promoter (Figure 7C). When the predictive binding sites on SLC1A5 promoter were mutated, KLF7 had no effect on the luciferase activity (Figure 7C). These results suggest that KLF7 transcriptionally activates SLC1A5 in HCC cells. Thus, it is necessary to illustrate the role of SLC1A5 in HCC and its contribution in KLF7‐triggered tumorigenesis. To address this question, we overexpressed SLC1A5 in shCtrl and shKLF7 SKHEP1 and Huh7 cells. SLC1A5 overexpression did not affect the expression of KLF7 and resulted in up‐regulation of SLC1A5 in shCtrl and shKLF7 cells (Figure 7D). Overexpression of SLC1A5 alone increased the concentration of Trp and 5‐HT, the growth and migration ability of HCC cells (Figure 7E–H), suggesting the oncogenic function of SLC1A5. KLF7 knockdown decreased Trp and 5‐HT concentrations in SKHEP1 and Huh7 cells, while SLC1A5 overexpression significantly increased their content (Figure 7E). These results indicated that KLF7 regulates Trp metabolism by targeting SLC1A5. Furthermore, we also found that SLC1A5 overexpression could recover the proliferation and migration ability of HCC cells inhibited by KLF7 downregulation (Figure 7F–H). Pearson's correlation analyses indicated that the expression of KLF7 was positively correlated with that of SLC1A5 expression in HCC samples based on The Cancer Genome Atlas (TCGA) database (*r* = 0.27, Figure 7I). IHC staining results suggested that there was a positive correlation between KLF7 and SLC1A5 in human HCC samples (Figure S2 and Table S1). Collectively, KLF7 activates tryptophan metabolism by up‐regulating SLC1A5.

## 4 Discussion

Tumours utilise Trp and its metabolites to drive their development and evade host defences. While increasing capture of Trp through the up‐regulation of Trp transporter gene, core enzymes of Trp degradation are also up‐regulated, and others are down‐regulated [29]. Trp is one of the eight basic amino acids, and a large proportion of cellular Trp is converted into the serotonin and kynurenine pathways, which are regulated by MYC up‐regulation of Trp importers SLC1A5 and SLC7A5 [19, 30]. In this study, we found that Trp metabolism was suppressed in KLF7‐knockdown HCC cells using RNA sequencing and metabonomic analysis.

In humans, there are 458 proteins of the Solute Carrier membrane‐bound transporter 65 families [31] with significant physiological effects, which are key to metabolic activation and cellular function [32]. SLC1A5 promotes the transport of glutamine leucine and Trp in cancer cells and controlled by mTORC1 and MYC signalling pathway [19, 33]. SLC7A5 is a membrane transporter of essential amino acids, including leucine, phenylalanine, and Trp, and is expressed in the brain endothelium, placenta, spleen, and bone marrow [34, 35]. Tryptophan hydroxylase (TPH) has two isoforms (TPH1 and TPH2), which catalyses the production of L‐5‐hydroxytryptophan (5‐HTP) from Trp and the decarboxylation of 5‐HTP to produce serotonin (5‐hydroxytryptamine, 5‐HT) [27]. Serotonin, a neurotransmitter and vasoconstrictor, is synthesised in the central and peripheral systems as a central signalling molecule involved in tumorigenesis, development, and metastasis of human cancers [36]. To further define the molecular mechanisms of Trp metabolism that participate in KLF7‐regulated HCC growth, we measured the expression of three markers (SLC1A5, SLC7A5, and TPH1), which are related to Trp metabolism. The results of RT‐qPCR and western blotting showed that the mRNA and protein levels of SLC1A5, SLC7A5, and TPH1 decreased significantly after KLF7 knockdown, while they increased greatly after KLF7 overexpression. Furthermore, we directly measured the contents of Trp and serotonin (5‐HT) in HCC cells treated with KLF7. The results showed that inhibiting KLF7 inhibited Trp and 5‐HT concentrations, while ectopic expression of KLF7 induced Trp and 5‐HT levels, indicating that KLF7 promotes Trp uptake and intracellular 5‐HT metabolism in HCC. These results suggested that KLF7 facilitated the abundance of trp via regulating the expression of SLC1A5, SLC7A5, and TPH1. Previous studies showed that SLC1A5‐mediated glutamine metabolism was critical for carcinogenesis [37, 38]. L‐aromatic amino acid decarboxylase (AADC) is another important enzyme catalysing the metabolism of 5‐HT [39]. The significance of glutamine metabolism and AADC in KLF7‐triggered HCC development should be investigated in the follow‐up study.

Trp metabolism plays an important role in the regulation of immunity, neuronal effects, and intestinal homeostasis. Imbalances in Trp metabolism have been observed in disorders ranging from cancer to neurodegenerative diseases, motivating the interest to targeting the pathways or limiting enzymes of Trp metabolism for the treatment of these diseases [17]. Chen et al. [40] revealed that an imbalance in the composition of the intestinal microflora drives the initiation of liver cancer by modulating Trp metabolism through up‐regulation of the sterol regulatory element binding protein 2. Ala [41] summarised that Trp metabolites affect the immune system and modulates inflammatory bowel disease and colorectal cancer. In HCC, Xue et al. [42] found that Trp metabolism‐related genes could be applied to forecast prognosis and instruct immunotherapy among patients with hepatocellular carcinoma. Li et al. [43] showed that Trp 2,3‐dioxygenase (TDO2) drove emergency medical technician (Epithelial–mesenchymal transition) from HCC through the Kyn‐AhR pathway. TDO2 could be a target of miRNAs in HCC that promoted tumour cell proliferation, metastasis, and invasion [44]. Furthermore, KLFs were involved in Trp metabolism [45, 46]. Therefore, we wondered whether KLF7 promoted the progression of HCC through Trp metabolism. To answer this question, we detected HCC cell propagation and migration in vitro and tumour development in vivo under KLF7 knockdown and treatment with serotonin or its inhibitor. In vitro, serotonin could improve cell propagation and migration capacity of HCC cells which were inhibited by KLF7 knockdown. Additionally, inhibitor sertraline or cultured cells in DMEM/F‐12 medium lacking Trp reversed the oncogenic function of KLF7. In vivo, the mice exposed to double tryptophan diet had a larger tumour size and weight, whereas mice with KLF7 knockdown exhibited smaller tumours. The results of Ki‐67 expression were also consistent with those of the tumour growth curves and tumour weights. These results showed that KLF7 drove the progression of HCC through the intake of Trp, while whether KLF7‐mediated trp import contributed to HCC development through the metabolism pathway of serotonin and the in vivo role of KLF7‐mediated trp metabolism in HCC metastasis should be determined in the follow up study.

KLFs can act as transcriptional activators or repressors by interaction with GC‐rich consensus in target genes, such as 5′‐CACCC‐3′ DNA sequences [9]. There are two domains in the SLC1A5 protomer, a transport domain and a scaffold domain linked by the extracellular loop region 2 [47]. SLC1A5 interacts with and may be regulated by the discoid protein domain receptor 1 in the progression of HCC. DDR1 is a special type of tyrosine kinase of the transmembrane receptor [48]. SLC1A5 plays a significant role in cancer cell metabolism, development, and propagation, and has received attention as a pharmacological target to block cancer cell development and survival [49]. SLC1A5, an independent risk factor for HBV‐related HCC, is associated with the poor prognosis, disease progression, and an immunosuppressive microenvironment [50]. In this study, we predicted that there were several KLF7's binding sequences in the promoter of SLC1A5, SLC7A5 and TPH1 genes. Therefore, KLF7 may potentiate the expression these genes through directly interaction with their promoter, leading to activation of the transcription. To validate the mechanism, we conducted ChIP‐qPCR and luciferase experiments focusing on SLC1A5 gene. We identified the interaction between KLF7 and the SLC1A5 promoter by ChIP‐qPCR assay. Luciferase reporter results showed that KLF7 potentiated the transcription activity when HCC cells expressed the widetype, but not the mutated promoter sequence of SLC1A5 gene. In addition, the level of SLC1A5 protein in HCC cells was regulated by KLF7, and overexpression of SLC1A5 restored the ability for cell proliferation and migration that decreased subsequent to KLF7 knockdown. Pearson's correlation analyses and IHC staining results indicated a positive association between KLF7 and SLC1A5 expression in HCC samples. Collectively, we demonstrated that KLF7 transcriptionally activated SLC1A5 in HCC cells and HCC patients. In the future, we will deeply illustrate the precise mechanisms how KLF7 regulates SLC7A5 and TPH1.

## 5 Conclusions

In summary, this study showed that KLF7 promoted the development of HCC through Trp metabolism and identified the involvement of new axis of KLF7/SLC1A5 in HCC, which may represent therapeutic markers for HCC.

## Author Contributions

**Bao Chai:** formal analysis (equal), investigation (equal), methodology (equal), writing – review and editing (equal). **Anhong Zhang:** data curation (equal), formal analysis (equal), investigation (equal), writing – review and editing (equal). **Yang Liu:** data curation (equal), formal analysis (equal), investigation (equal), writing – review and editing (equal). **Xi Zhang:** data curation (equal), formal analysis (equal), investigation (equal), writing – review and editing (equal). **Pengzhou Kong:** formal analysis (equal), investigation (equal), software (equal), writing – review and editing (equal). **Zhuowei Zhang:** formal analysis (equal), investigation (equal), software (equal), writing – review and editing (equal). **Yarong Guo:** conceptualization (equal), funding acquisition (equal), methodology (equal), writing – review and editing (equal).

## Ethics Statement

The animal study was approved by the Ethics Committee of the First Affiliated Hospital of Shanxi Medical University.

## Conflicts of Interest

The authors declare no conflicts of interests.

## Supporting information


Appendix S1.

## Data Availability

The data that support the findings of this study are available from the corresponding author upon reasonable request.
