# Supplementary material for: KLF7 Promotes Hepatocellular Carcinoma Progression Through Regulating SLC1A5‐Mediated Tryptophan Metabolism
Source: J Cell Mol Med. 2024 Dec 8;28(23):e70245. doi: 10.1111/jcmm.70245 (PMC11625504; doi:10.1111/jcmm.70245)
Supplement: Supplementary file 1 — Appendix S1. [file JCMM-28-e70245-s001.docx]

**
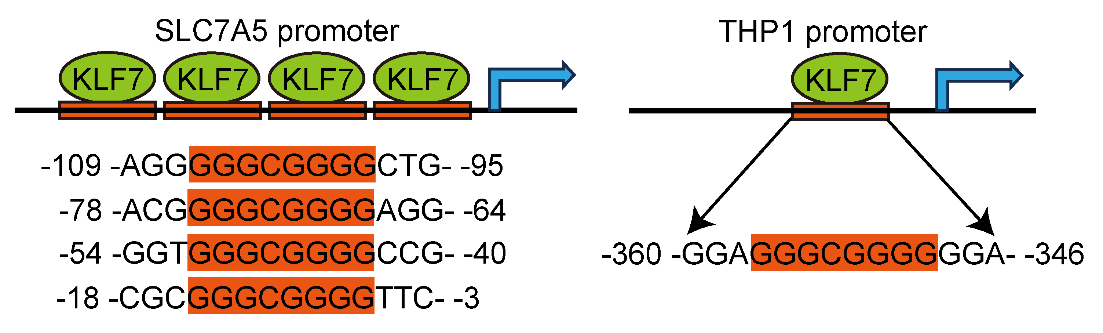
**

**Figure S1 The predictive binding sequence of SLC7A5 and THP1 promoter for KLF7.**

**
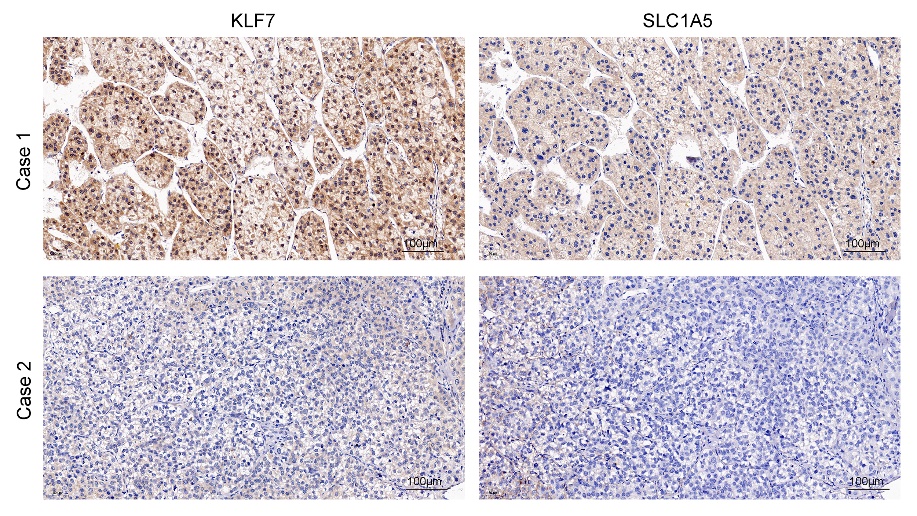
**

**Figure S2** IHC staining of KLF7 and SLC1A5 in HCC patients. IHC analysis of KLF7 and SLC1A5 in HCC tissues. Scale bar, 100 μm.

**Table S1** Spearman correlation analysis of expression between KLF7 and SLC1A5 in 30 HCC tissues by IHC.

|  | KLF13 | |  |
| --- | --- | --- | --- |
|  | rs | P value |  |
| SLC1A5 | 0.228 | 0.0076 |  |

r, Spearman correlation
